# Bibliographical Notices

**Published:** 1847-08

**Authors:** 


### BIBLIOGRAPHICAL NOTICES.

*The Principles and Practice of Ophthalmic Medicine and Surgery.* By T. Wharton Jones, F. R. S., Lecturer on Anatomy, Physiology, and Pathology, at the Chareing Cross Hospital, &c. &c. With one hundred and two illustrations. Edited by Isaac Hays, M. D., Surgeon to Will's Hospital, etc. 1 vol. 12 mo. Lea & Blanchard publishers, Philadelphia, 1847. p. p. 509.

This is one of a series of most excellent Medical and Surgical Manuals, or text books, and to us it is not the least valuable of them all. A well digested *text book* upon diseases of the eye has been a desideratum which both teachers and pupils have felt, and which is now happily supplied. The book is concise, clear, and well brought up to the discoveries and improvements of the day ; and in no department have such rapid advances been recently made, as in Ophthalmic Surgery, and especially among the Germans, under the practical investigations of such men Ammon, Chelius, Graefe, Himly, Juengken,Ructe, and others. The author himself, also, has added considerable original matter from his own extensive experience, and has admirably arranged the whole.

In the introduction we are furnished with a brief history of Ophthalmic Medicine and Surgery.

The text is divided into 11 chapters, and the chapters into sections and sub-sections, the sub-sections numbering in all 2688, which, with a copious index, renders a reference to any passage in the book exceedingly easy.

As an illustration of the anthor's method, we have taken at hazard the following general remarks upon the subject of *Eye-salves:*

"*Eye-salves*—Salves are applied to the borders of the eyelids, or to the whole conjunctival surface. In the former case only, should the patient or his attendants be intrusted with the application. In the latter case, more discrimination, as well as tact, being required, the surgeon should apply the salve himself.

Before applying a salve to the edges of the eyelids, all incrustations of matter about the roots of the eyelashes must be removed. This is done by first rubbing the part with fresh butter or lard, and after a while bathing it with tepid water; the incrusting matter is thus softened, and may readily be separated with the finger-nail or head of a pin.

The anointing of the edges of the eyelids may be performed by means of a hair pencil, or simply with the point of the finger. The eyelid being held slightly everted, the salve is applied along its border to the mouths of the Meibomian follicles, then smeared outside the insertion of the eyelashes, and afterwards carefully rubbed in at their roots, the eyelids being at the time kept gently closed.

When a salve is to be applied to the whole surface of the conjunctiva, a piece the size of a split pea is to be taken up on the point of a probe, or on the point of the nail of the little finger, and insinuated under the upper eyelid, while this is drawn forward from contact with the eyeball. When the salve is fairly in the eye, the upper eyelid is to be gently drawn down, and rubbed over the eyeball with the finger for a minute or two, in order to diffuse the salve, now melted by the heat of the eye. between the eyelids and eyeball, and consequently all over the conjunctiva.

If the palpebral conjunctiva be the part on which the salve is principally to exert its action, the application may be limited to it, in which case the pain is less severe than in the former. Having everted the eyelids, the exposed conjunctival surface is to be rubbed with the salve either by means of a hair-pencil or the point of the finger.

Salves are sometimes applied to the lachrymal passages, by smearing the meshes, cat-gut strings, &c., of which are introduced into the nasal duct, whether from the nose or through an external opening into the lachrymal sac.

In regard to the application of a strong salve to the eye, it is necessary here to give a caution, viz., not to insert it in a lump within the lower eyelid and leave it there. I have seen the conjunctiva of the inferior palpebral sinus in a sloughy state from a lump of nitrate of silver ointment having been put in, and no care taken to diffuse it by rubbing the eyelid over the eyeball. In the case of ointment inserted under the upper eyelid, the natural motions of the part will make up in some degree for the neglect of the surgeon.

Examples of eye-salves.

R—Oxid. hydrarg. rubri bene lævigat. gr. iii—vi—xv.

Axungiæ præparat. ʒij.

Misce accuratissime: ft. unguentum ophthalmicum.

The two weaker forms are used for anointing the edges of the eyelids, and may be entrusted to the patient. The stronger form should be applied only by the surgeon himself. Applied to the whole conjuctival surface it is found a very efficient remedy in various inflammations of the conjunctiva, and ulcers, specks, &c., of the cornea. It is less severe in its operation than the nitrate of silver ointment.

The citrine ointment of the pharmacopœias, diluted with three or more parts of lard and oil, is a useful application to the edges of the eyelids.

R—Argent. nitrat. gr. iii—x.
Aq. destillat. q. s. ad solvend. nitrat.
Unguent. cetacei ʒj.
Prius solvatur nitras; dein misceatur accuratissime solutio cum unguento.

This ointment has been used with much success not only in chronic, but also in cutting short acute inflammations of the conjunctiva; but the pain it causes is very severe.

The following is what is known by the name of Janin's ointment for the eyes.

R—Præcipitat. alb. gr. xv.

Tutiæ præparat.

Boli Armen. ppt. aa. ʒfs.

Adip. suilli ʒi—ʒij.

M. exactissime: ft. ungt. ophthalmicum.

It is of great consequence that eye-salves should contain no gritty particles; the powders entering into their composition, therefore, should first be reduced by trituration to as impalpable a state as possible, and then carefully levigated with a little water or oil, previously to being mixed with the excipient. Substances soluble in a small quantity of fluid, such as the nitrate of silver and sulphates of zinc and copper, may be dissolved. When camphor enters as an ingredient into an eye-salve, it should first be dissolved in a fixed oil. The excipient best adapted for eye-salves is prepared lard, simple cerate, or spermaceti ointment.

That nothing might be left which could render the work complete, an excellent glossary is appended, in which the student will find, not only the definition, but also the root of every technical employed. In short, let us say to the reader, it is a book—

"Which if you do open
To readen and copen
You will be unwilling
For many a shilling
To part with the profit
That you shall have of it."

F. H. H.

---

*The Gas War.*—We have received another pamphlet on the subject of the Gas discovery. The author is Edward Warren, of Boston, Mass. The object is to establish the claims of Dr. Morton to the discovery. We see nothing in it to lead us to alter our views as expressed in our last number. The contest appears to be growing warmer, and we are sorry to perceive in the pamphlet referred to, personalities which are not relevant to the matter at issue.

Since the above was written, we notice in the Boston Medical and Surgical Journal a note from Dr. Edward Warren, well known from his valuable contributions to Medical Journals, stating that he is not the author of the pamphlet referred to, but a non-medical gentleman of the same name who is connected in the business of the patent with Morton. We had supposed the author to be Dr. Warren ; we are happy to be corrected,and, in justice to that gentlemen, to obviate in so far as we can the same error in others.

---

*The Medical Students' Vade Mecum, or Manual of Examinations*, upon Anatomy, Physiology, Chemistry, Materia Medica, Surgery, Obstetrics, Practice of Medicine, (including physical diagnosis and diseases of the skin) and Poisons.

Second Edition, revised and greatly enlarged, by Geo. Mendenhall, M. D., Lecturer on Pathology in the Medical Institute of

Cincinnati and member of the Philadelphia Medical Society, &c. &c., Philadelphia, Lindsay & Blakiston, 1845, 12 mo. p. p. 474.

We cannot concur in the opinion expressed by some of our contemporaries, that works of this character are devoid of utility. If devoted to the purposes for which they are prepared, viz: to refresh the memory, and aid the retention of important facts, we conceive that they may prove convenient and serviceable both to the student and practitioner. They are by no means to take the place of extended treatises, but to serve as aids in the reviewal of knowledge acquired from legitimate sources. For this end we think they may safely and properly be commended.

The work by Dr. Mendenhall has already secured favor, as evidenced by the speedy exhaustion of a former edition. The present edition has been carefully revised and improved, and about one hundred and fifty pages of new matter added. It is therefore still more deserving of approbation and success, which it will doubtless receive.

---

*Medical Botany, or Descriptions of the more important plants used in Medicine, with their history, properties, and mode of administration.* By R. Eglesfield Griffith, M. D., Member of the American philosophical Society; of the Academy of Natural Science, of Philadelphia, etc., etc. Scire potestates herbarum usumque medendi. Æneid xii. 396, with upwards of three hundred illustrations. Philadelphia, Lea & Blanchard, 1847. 8vo. pp. 704.

The objects and plan of this work will be understood from the following quotations from the preface:—

"The student who wishes to obtain correct systematical descriptions of the various plants employed in medicine, must either consult a long series of expensive books, or be content with the short notices to be met with in the usual treatises on the Materia Medica, and hence the important subject of Medical Botany has been almost wholly neglected in this country. To supply this want, has been the aim of the author in the present publication, which, whilst it gives a general view of the Vegetable Materia Medica, is still sufficiently cheap to be within the reach of all.

It may be asked, will not the excellent works of Pereira, Royle, Ballard and Garrod, and Drs. Wood and Bache, supply all the botanical knowledge that is required by the student? We think not, the intention of the authors of these treatises is rather to present to their readers an account of medicinal articles, and of their composition and uses, than to dwell on the characters and history of those derived from the vegetable kingdom; at the same time, they all contain much that is interesting and important on these topics. The work now offered to the profession is intended as a companion to the more practical treatises, and also to convey such information on the systematic classification, characters, and history of Medical Plants as has been necessarily omitted in the publications alluded to.

To render the publication more generally useful, especially to those readers who are not conversant with Botany, a short Introduction on the Structure and Composition of Plants has been prefixed, with a copious Glossary of terms, and a Conspectus of the Natural Orders of Plants containing remedial substances; these it is hoped will add to the value of the work, whilst they do not materially swell its bulk.

In the present state of Botanical Science, the most eminent authorities disagree as regards the best mode of arranging the natural orders, or their exact relations with each other. In this country, the classification of De Candolle is best known, more especially as modified by Drs. Torrey and Gray. By some, however, that of Lindley is preferred, whilst others prefer the system of Jussieu, &c. That adopted is founded on the views of De Candolle, but modified by those of Lindley, Gray, and others. The groups into which the orders have been divided, are mainly those suggested by the two latter authorities, but altered in some respects according to the views of the author.

In so far as we are capable of judging, the work is well adapted to fulfil the purposes for which it is designed, and will be found highly convenient and useful, as a work of reference, for the medical practitioner. The wood cuts are respectable, and the typography excellent.

